# Some Hints on the Preparation and Mounting of Microscopic Objects

**Published:** 1882-02

**Authors:** W. H. Walmsley


### SOME HINTS ON THE PREPARATION AND MOUNTING OF MICROSCOPIC OBJECTS.

BY W. H. WALMSLEY.

So much has been written and published on this well worn subject, that it would seem almost superfluous, if not presumptuous, in me to attempt to add thereto. But recollections of the many failures, in my early attempts in years long since gone by, of the wastage of time and materials incurred, and the unsatisfactory knowledge gleaned from books, impel me to jot down for the benefit of others, the results of actual experience in this work.

Whilst by no means asserting that the processes to be described are *the best*, I would say that I have found them to be uniformly satisfactory, yielding always the desired results, and that all have stood the tests of actual use and experience. I shall give nothing that I do not use in my daily work; and shall not state what "my friend Smith" *says* "is his process," or that "I am told Mr. Jones does this and that." Smith's and Jones' processes *may* be vastly superior to those I shall give, but not having tested, I shall not speak of them, my intention being to give simply and succinctly as possible, my methods of preparing and mounting ordinary objects of interest; which may prove of use to many a beginner in this fascinating pursuit.

Nearly all microscopic preparations are mounted in one of three ways: in balsam or other resinous media; in air in the dry way, and in aqueous or other fluids. Of these methods I shall proceed to speak first of balsam mounts, the *essential* materials for which work are as follows:

A bottle or tube of pure filtered Canada balsam; a bottle each of 95° alcohol, pure benzole, oil of cloves, and liquor potassæ (the latter with glass stopper); a pair of fine *curved* forceps, which should be nickel-plated; another of fine dissecting scissors, and a small dissecting knife; two needles in handles; a few small, red, sable brushes; one large camel's hair brush; a glass pomatum jar; nest of porcelain dishes, and a few watch glasses; a wide-mouth 8 oz. vial, with glass stopper; small glass rod; some pieces of very fine brass wire; glass slips and covers, with suitable labels; and a small bell-glass.

To these may be added the following *non-essential*, but very convenient articles: A capped bottle, with glass rod, for containing the balsam; a small, brass table with spirit lamp; a turn table; porcelain mounting plate (hereafter to be described); magnifying glass on stand, with elongating arm; white zinc cement, shellac ditto, and colored fluid for ornamental ringing; a bottle of absolute alcohol; and a writing diamond. The *luxuries* may be mentioned under the head of a well made self-centering turn table; a hot water drying oven; fine spring scissors; an assortment of dissecting needles, hooks, scissors, and knives; and a pair of binocular magnifiers, mounted upon a firm stand, with focussing adjustment. But all the processes of mounting to be here named may be performed with the tools and materials mentioned under the head of *essentials.*

Having thus started in business with our capital of tools and materials, let us proceed to put them to the test of actual use. And I can provide no better subject for a beginning than a common blow fly, or an ordinary house fly, either of which will afford material for several different processes of balsam mounting. A female should be selected, as the ovipositor, which usually contains some eggs, affords a most interesting and beautiful object.

Vivisection not being favored by the writer—first kill your fly in the most humane manner possible (chloroform is recommended; then proceed to clip the wings off close to the body, which, not needing any preparation, may at once be placed in alcohol, in one of the covered porcelain saucers. The legs, being cut off, should be placed in liquor potassæ, which should be contained in the glass pomatum jar with a cover. By gently pressing the abdomen, the ovipositor will protrude to its full length, and should then be cut off close to the body, and also placed in the liquor potassæ. The tongue should be pressed out in like manner, and when found (under the magnifying glass) to protrude to the full extent, with all its parts, should in like manner be cut off and follow the legs and ovipositor. Then the abdomen may be cut open with the scissors, the viscera washed out with the small sable brushes and water, and the skin or epidermis containing the spiracles be placed in the liquor potassæ. The trachea and eyes, requiring different treatment to that we are now pursuing, will not be followed further at present.

The length of time necessary for the various parts to remain in the liquor potassæ varies materially. Thus an hour, or at the most two, will suffice for the tongue and ovipositor, whereas the legs and epidermis will require an immersion of not less than one or two days. Great care should be taken to remove them before too much color is abstracted, as the beauty of a preparation is quite lost if it be pale and colorless. A good rule is to remove these parts from the liquor as soon as they assume a lightish brown appearance; placing them in water, and carefully washing and brushing them with the sable brushes. One of the nest saucers will be found a most convenient vessel for doing this in. They should then be transferred to a glass slip, taking care (with the tongue) so to spread it out with the needles as to show the lobes and false trachea, and (with the feet) to show the hairy pads. When properly spread out, place another glass slip over them so that they are pressed flat between the two, and wrap tightly with a piece of fine brass wire, which for this purpose should be cut in lengths of 10 or 12 inches. The wire is recommended, rather than thread of any kind, because there are no fibres to become entangled with the specimen, and thus mar its beauty; and it may be used many times over. The slips thus wrapped should then be dropped into a vessel of water, and left for some hours. On being taken from the water and the wire removed, the slips should again be placed in a saucer or small plate of water, and carefully separated to avoid marring or injuring the specimen. These should then be gently, but thoroughly, washed and brushed, to remove every remnant of the liquor potassæ or dirt that may have adhered to them, and dropped for a few moments into alcohol; one of the nest saucers again forming a convenient vessel for this purpose. The slips of glass having meanwhile been wiped clean and dry, the objects are again to be transferred to one of them, covered with the other, wrapped with the wire and dropped into alcohol, which for this purpose should be contained in the wide mouthed bottle with glass stopper. And here they may safely rest until we are nearly ready for the final operation of mounting; be it the next day, or year, matters not, as the alcohol will not alter or bleach them.

The final work to be done upon our specimens, preparatory to mounting, is transferring from the alcohol to oil of cloves, which is a substantial repetition of the previous transfer from water to alcohol. The slips of glass taken from the bottle, and the wire removed, are to be placed in a saucer containing alcohol, and gently separated, to avoid injury to the specimens, which are now to receive their final brushing. If we have provided ourselves with absolute alcohol, a short immersion in the same, in a watch glass is advantageous, but not absolutely necessary. And now having poured a small quantity of pure oil of cloves into one of the porcelain nest saucers, we carefully transfer the tongue, feet, etc., to the same, not forgetting the wings (which all this time have lain quietly in the alcohol, as originally placed, not having needed any of these complicated manipulations), immediately replacing the cover to exclude dust.

It will be observed that I rigorously exclude the turpentine in all forms from my work. I have ever found it a most unsatisfactory medium, foul smelling, sticky, and rendering all tissues immersed in it stiff and brittle. Oil of cloves, on the contrary, is in all respects a most admirable medium, rendering all tissues and substances fully as clear as turpentine; is agreeable to the sense of smell, does not stiffen anything immersed in it, and is perfectly miscible with balsam or damar.

After this digression, and whilst our specimens are clearing up in the oil, let us see to our glass slips and covers, and to the balsam in which the former are to be mounted. Very many processes for cleaning the slips and covers have been given to the world by various writers, and probably they are all good. I give only my own, which I have used for many years with entire satisfaction, and therefore can confidently recommend it. The slips (which should be smooth edged) are placed in a basin with *hot* water and good soap, and wiped dry with a soft towel, after being thoroughly washed and rinsed. They are then placed in a drawer, and are ready to use at any future time, merely requiring to be brushed off with the large camel's hair pencil when used. The thin covers (which should *always* be circles, and not squares, as making neater and more readily finished mounts,) are dropped one by one in a glass tumbler, containing sulphuric acid, and allowed to remain there for some hours. The acid is then poured off, and water *carefully* added, which in its turn is decanted and replaced with fresh water, the whole contents of the glass being freely agitated until every trace of the acid is removed. One of the glass pomatum jars is now to be partially filled with alcohol, and the thin covers placed therein to remain until wanted for use, when they can be removed with the forceps, and a slight wiping with an old, soft linen handkerchief will leave them brilliantly clean.

My own preference is for absolutely pure filtered balsam as affording the most satisfactory results in all sorts of mounts, but that from which the spirits of turpentine has been expelled by heat, and replaced by cloroform or pure benzole, answers a most excellent purpose, whilst the many damar mediums are also good. But following the plan upon which this article was begun, of giving only those processes which I habitually practice, and know to be satisfactory, I shall confine myself to pure balsam alone. The great secret in its successful use, is to have it of just the proper consistency, neither hard enough to resist the thrusting of a fly's wing into a drop of it when placed upon the slide, or so limpid as to spread and run when so placed. If too thick, a little chloroform carefully stirred into the bottle containing the balsam will reduce it to proper consistency; if too thin, a covering of cotton cloth should be placed over the mouth to exclude dust, and the bottle put in a warm place for a day or two. My own plan is to put it—when found just right---into collapsible tubes, from which the proper amount can be squeezed out upon the slide, and in which it will remain of the same consistency until the last drop is used; but most persons will prefer to dip it from a wide mouthed bottle with a small glass rod, and to such I would strongly recommend the capped bottle, which I have named under the head of *non-essentials*, as it excludes all dust, allows the rod to remain in the balsam when not in use, and prevents any foreign particles from falling into the latter, as must be e case in all bottles closed with a cork.

And now we have brought our work down almost to the final operation. Our specimens are soaking in oil of cloves waiting to be mounted, our glass is clean and bright, our balsam in its bottle, our forceps and needles lying in their places ready for instant use.

What more do we need? Only a lamp or other convenient method close at hand for warming the slide and cover; and some mode of *centering* the object before applying the cover. The latter *may* be done by laying a slide on a sheet of white paper or card board, and drawing its outline with a pencil, and on removing the slide making a mark exactly in the center of this drawing. Of course, when the slide is replaced, this mark will apparently be in *its* center, and thus the balsam and object can be accurately placed in proper position. The principal objection to this method is, that the slide is apt to slip out of place, and thus to render accurate centering almost impossible. A most admirable contrivance for this purpose, however, is the "porcelain mounting plate," named under the head of *non-essentials.* This is made of a photographic porcelain plate, 3x5 inches, ground flat upon one side, on which are cemented at right angles to each other, two small strips of glass, the one three inches in length, and the other one inch. A mark is then made upon the plate with a pencil, exactly $1\frac{1}{2}$ inches from the strip, and $\frac{1}{2}$ an inch from the other, so that when the ordinary glass slip is placed upon the plate, with one end in contact with the two strips, this mark is seen exactly in the center. The slip is held firmly in place, and the white surface of the plate serves admirably as a background upon which to arrange the object.

At last we seem to be really done with all our preliminary work, and ready for mounting. But wait another moment. Though as before stated, the oil of cloves is perfectly miscible with the balsam, and a specimen may be transferred directly from the one to the other, it is a very slow drier, and an object so mounted might be months in hardening sufficiently to handle, even if the utmost precaution be taken to drain off all the superfluous oil. Fortunately we have an excellent remedy for this trouble close at hand. Pouring a small quantity of benzole into a watch glass, we place in it one of the specimens, say a wing, and immediately cover it with the small bell glass to exclude dust, and prevent the evaporation of the benzole, which is exceedingly volatile. And now at last we are ready for the mounting.

One of the cleaned slips having been placed upon the mounting plate, and its surface dusted off with the brush, a drop of balsam of exactly the proper dimensions, is to be placed in its center, indicated by the mark upon the plate. And here, practice alone must be our guide, for the amount must be varied according to the diameter of the covering glass and the thickness of the object. In mounting our present specimens, say with covers of $\frac{5}{8}$ inch diameter, it will be found that more of the balsam will be required to fill up evenly to the edge of the cover with one of the legs than with a wing. The great aim should be to get exactly the right amount dropped on the slide at first, so that it will fill to the edge of the cover when the latter is pressed down, without any excess exuding, or any additional filling being required. No rule can be given for doing this; only continuous practice can give one the necessary skill; but it is worth trying for, since a mount thus made possesses an artistic appearance, which cannot be equalled by any other means. But to return to our muttons. The drop of balsam having been placed in the center of the slide, the wing is taken from the benzole with the forceps, and thrust into it---the balsam---gently, but firmly; and then with one of the needles it is to be pushed down upon the slide, (into firm contact with its surface, in fact,) and arranged in proper position. Now mark this point carefully. If the object be left suspended in the drop of balsam, as soon as the covering glass is applied, it will float out of position, and much valuable time, and more patience be consumed in getting it back, together with the added risk of destroying it in the attempt. But if it be placed in close contact with the surface of the slide, it will become firmly fixed. The forceps having been wiped clean (it is still better to have a second and heavier pair for this purpose), one of the cleansed covers is taken up by them, slightly warmed over the lamp, and gently laid upon the balsam, which will spread out under its warmth, if the operation be successfully performed, as the cover settles down to its level. This can be aided by carefully warming the slide over the lamp, holding it in a perfectly level position, and the warming should be continued until the fluid balsam has reached the edge of the cover all around, when the slide should be set aside in a moderately warm place for say 24 hours. On no account should the cover be touched or pressed down at this time, and no notice should be taken of any bubbles that may appear, as they will move out to the edges and depart of their own accord. On the following day the slide may be again very gently warmed, and the cover very slightly pressed down with the forceps, after which, if we possess the luxury of a drying oven, we will place it therein, and by the aid of a small kerosene lamp, maintain a temperature of about 120° Fahrenheit for a week or ten days, when the preparation may be taken out to remain, we trust, "a thing of beauty and a joy forever."

The same process that we have followed to its end with the wing, suffices for all the other portions of our fly, and, indeed, for all specimens not thick enough to require a cell; for these special directions will be given in a future article.

If our work has been entirely successful, the slide is now finished, with the exception of labeling. But if (as is more likely), there was an excess of balsam, which has exuded from beneath the cover, it must be cleaned off with a knife, and being by this time hard and resinous, this is very readily done, after which a little soap and water will remove all traces of it. Most persons will prefer to finish their slides with a ring of cement, and for this purpose a turn-table, which I have enumerated among the *non-essentials* for balsam mounts, must be provided. The slide having been accurately centered, a ring of the shellac cement is to be applied, followed by successive ones of white zinc, until the space between the surfaces of the slide and covering glass is quite filled up, care being taken to allow each coat to harden before applying a fresh one, which is rapidly accomplished with this invaluable cement. A narrow ring of some bright color makes a pleasing finish, and can be readily made by adding to the ordinary damar medium a sufficient quantity of any desired color, ground in a little oil. All these cements should be applied by small *red sable* brushes, and the best mode of using them is to have the bottles in which they are contained filled with long tapering corks, into the under side of which the brushes are to be fastened. They are thus always immersed in the cement when not in use, and never can become stiff and dry; whilst they undergo no apparent deterioration. I am now using in my white zinc bottle the same brush that I placed there six years ago, during which time it has been employed to ring many thousands of slides, and is now as "good as new." The long cork forms a very convenient handle for the brush, and if care be taken to wipe it and the neck of the bottle with alcohol, occasionally, they will never stick together.

It is to be noted that the flattening between glass slips, etc., is only necessary in the case of objects of considerable thickness, such as the fly's leg we have been preparing. All *thin* objects, as sections of animal or vegetable tissues, etc., may be carried through the alcohol and subsequent stages, in the same manner as that pursued with the fly's wing; or, if perfectly *dry*, may be at once immersed in the oil of cloves, or even mounted in the balsam, without previous soaking in anything.

And now, having brought our balsam mounts to a successful completion, I must also make an ending of the present paper. In future ones (if this be well received), I propose to give some practical hints on mounting in the dry way, both opaque and transparent objects suited to that method of preparation; also on fluid mounts of various sorts, and possibly others on the double staining of vegetable tissues.

Should any of my readers desire to see a balsam mount prepared according to the foregoing directions, I shall be pleased to exchange with him or her for any well prepared original slide.—*The Microscope.*

